# Supplementary material for: Colorectal Cancer and Onset of Anxiety and Depression: A Systematic Review and Meta-Analysis
Source: Curr Oncol. 2022 Nov 15;29(11):8751–66. doi: 10.3390/curroncol29110689 (PMC9689519; doi:10.3390/curroncol29110689)
Supplement: Supplementary file 1 [file curroncol-29-00689-s001.zip › curroncol-1969632-supplementary.pdf]

Current Oncology

MDPI

Systematic Review

Colorectal Cancer and Onset of Anxiety and Depression: A Systematic Review and Meta-Analysis

Vicki Cheng [1,2], Niki Oveisi [1,2], Helen McTaggart-Cowan [3,4], Jonathan M. Loree [5,6], Rachel A. Murphy [3,7], Mary A. De Vera [1,2,8,*]

Table S2. EMBASE Ovid Search (1974 to March 01, 2022); updated search ran June 29, 2022.

Line	Searches	Results
1.	exp colon cancer/ or exp colon carcinoma/ or exp rectum cancer/ or exp colorectal cancer/ or exp rectum tumor/ or exp colon tumor/ or exp rectum carcinoma/	407988
2.	((colorectal or colon or rect*) adj3 (cancer* or neoplasm*)).mp. [mp=title, abstract, heading word, drug trade name, original title, device manufacturer, drug manufacturer, device trade name, keyword heading word, floating subheading word, candidate term word]	363402
3.	1 or 2	446177
4.	exp minor depression/ or exp "mixed anxiety and depression"/ or exp "Depression, Anxiety and Stress Scale"/ or exp "Depression, Anxiety and Stress Scale-21"/ or exp "mixed depression and dementia"/ or exp chronic depression/ or exp depression/ or exp adolescent depression/ or exp bipolar depression/ or exp "Hospital Anxiety and Depression Scale-Depression"/ or exp "Hospital Anxiety and Depression Scale-Anxiety"/ or exp depression assessment/ or exp "Hospital Anxiety and Depression Scale"/ or exp agitated depression/ or exp depression inventory/ or exp atypical depression/ or exp long term depression/ or exp late life depression/ or exp "mixed mania and depression"/ or exp Child Depression Inventory/ or exp major depression/ or exp Major Depression Inventory/	576624
5.	(depressive or depression or melancholia* or "involutional psychosis" or "involutional psychoses" or "dysthymic disorder*" or "premenstrual dysphoric disorder*" or "seasonal affective disorder*" or "seasonal mood disorder*").ti,ab.	583651
6.	4 or 5	780189
7.	exp social anxiety/ or exp generalized anxiety disorder/ or exp "Depression, Anxiety and Stress Scale-21"/ or exp Generalized Anxiety Disorder Scale/ or exp "Depression, Anxiety and Stress Scale"/ or exp "Hospital Anxiety and Depression Scale-Depression"/ or exp "mixed anxiety and depression"/ or exp anxiety assessment/ or exp anxiety disorder/ or exp Generalized Anxiety Disorder-7/ or exp anxiety/ or exp Generalized Anxiety Disorder-2/ or exp "Hospital Anxiety and Depression Scale-Anxiety"/ or exp "Hospital Anxiety and Depression Scale"/	499591
8.	(anxiety or agoraphobi* or "neurocirculatory asthenia" or "cardiac neuros*" or "hyperkinetic heart syndrome" or "neurotic disorder*" or psychoneurosis or psychoneuroses or neurosis or neuroses or "obsessive compulsive" or OCD or "anankastic personality" or "hoarding disorder*" or "obsessive hoarding" or "panic disorder*" or "panic attack*" or phobic or phobia* or claustrophobi* or "social anxiety disorder*").ti,ab.	357382
9.	7 or 8	568054
10.	6 or 9	1087988
11.	3 and 10	5653
12.	limit 11 to human	5339
13.	limit 12 to dc=20220228-20220629	219
14.	limit 13 to embase	160

Table S3. Ovid MEDLINE(R) and Epub Ahead of Print, In-Process, In-Data-Review & Other Non-Indexed Citations, Daily and Versions(R) Search <1946 to March 01, 2022; updated search ran June 29, 2022>.

Line	Searches	Results
1.	exp Colorectal Neoplasms/	221651
2.	((colorectal or colon or rect*) adj3 (cancer* or neoplasm*)).mp. [mp=title, abstract, original title, name of substance word, subject heading word, floating sub-heading word, keyword heading word, organism supplementary concept word, protocol supplementary concept word, rare disease supplementary concept word, unique identifier, synonyms]	239305
3.	1 or 2	281801
4.	exp "Depressive Disorder"/ or "Depression"/	240636
5.	(depressive or depression or melancholia* or "involutional psychosis" or "involutional psychoses" or "dysthymic disorder*" or "premenstrual dysphoric disorder*" or "seasonal affective disorder*" or "seasonal mood disorder*").mp.	511726
6.	4 or 5	511726
7.	exp "Anxiety Disorders"/ or Anxiety/	170251
8.	(anxiety or agoraphobi* or "neurocirculatory asthenia" or "cardiac neuros*" or "hyperkinetic heart syndrome" or "neurotic disorder*" or psychoneurosis or psychoneuroses or neurosis or neuroses or "obsessive compulsive" or OCD or "anankastic personality" or "hoarding disorder*" or "obsessive hoarding" or "panic disorder*" or "panic attack*" or phobic or phobia* or claustrophobi* or "social anxiety disorder*").mp.	322868
9.	7 or 8	322868
10.	6 or 9	699294
11.	3 and 10	1787
12.	limit 11 to dt=20220228-20220629	39

Table S4. Summary of anxiety and depression assessment methods and definitions.

Author, year	Outcome	Assessment Method	Case Definitions
Zhang, 2010	anxiety	[a]ICD-9 (code 300.0)	A diagnosis was assigned when an individual was diagnosed at least once for a disorder during a given year
	depression	[a]ICD-9 (codes 296.2, 296.3, 311, 300.4, 3090)	
Sun, 2017	anxiety	[a]ICD 9 (codes 300.0, 300.2, 300.3, 308.3, 309.81)	N/A
	depression	[a]ICD-9 (codes 296.2, 296.3, 300.4, and 311)	
Mols, 2018	anxiety	[b]HADS-A (7 items for anxiety)	Self-assesses levels of symptoms in the last week<br>The cut-off value for anxiety and depression symptoms is indicated by a score ≥ 8
	depression	[b]HADS-D (7 items for depression)	
Lloyd, 2019	anxiety	[a]ICD-9 (codes 5.1, 5.2, 5.4, 5.5, 5.5.1)	N/A
	depression	[a]ICD-9 (codes 5.8, 5.8.1, 5.8.2)	
Kjaer, 2021	depression	[a]ICD-9	First redeemed antidepressant prescription, or initial depression hospitalization or hospitalization for other major psychiatric events
Lee, 2021	anxiety	[a]ICD-9 (code 300.0)	[a]ICD-9 + at least 3 records of outpatient visits or 1 inpatient diagnosis during study period
	depression	[a]ICD-9 (codes 269.2, 269.3, 300.4, 311.X)	

Weissman, 2021	depression	[c]SNOMED-CT (“Depressive Disorder”; “Major Depressive Disorder”) and [a]ICD-10 (codes F54, F32.9, F33.0)	N/A
Howren, 2022	anxiety	[a]ICD-9 (codes 300.0, 300.2) and ICD-10 (codes F40-F41)	≥ 1 inpatient ICD code; or 2 outpatient ICD codes within a 2-year period
	depression	[a]ICD-9 (codes 296.2, 296.3, 296.5, 300.4, 309.x, 311.x) and ICD-10 (codes F20.4, F31.3-F31.5, F32.x, F33.x, F34.1, F41.2, F43.2)	≥ 1 inpatient ICD code; or 2 outpatient ICD codes within a 1-year period

[a]International Classification of Diseases; [b]Hospital Anxiety and Depression Scale; [c]Systemized Nomenclature of Medicine – Clinical Terms.

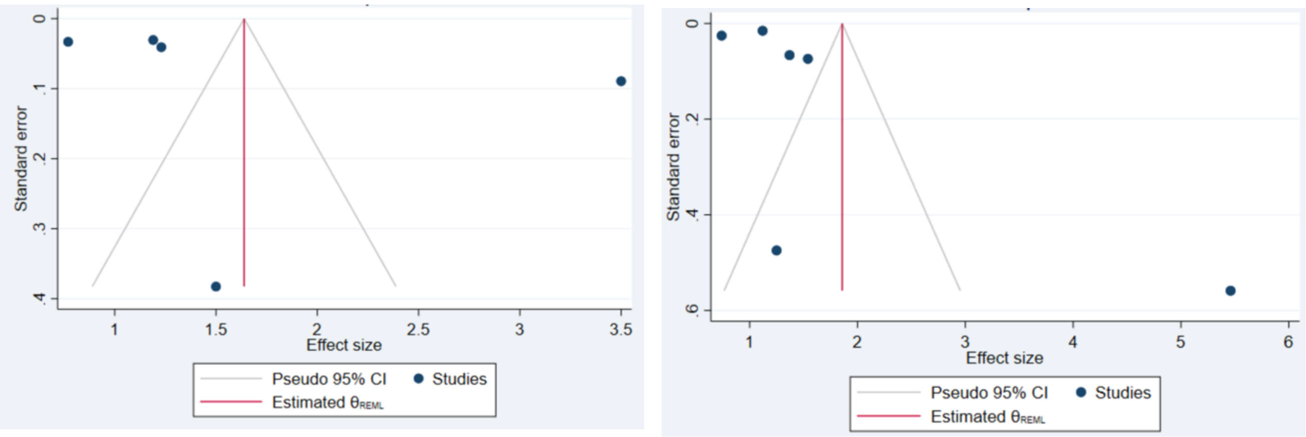

(**a**) (**b**)

Figure S1. Funnel plots of included studies in anxiety (**a**) and depression (**b**).
